# Beta-alanine provides partial protection against the development of symptoms driven by glucocorticoid excess

**DOI:** 10.1007/s00726-026-03536-y

**Published:** 2026-07-23

**Authors:** Samuel R. Heaselgrave, Minghao Deng, Gareth G. Lavery, Craig L. Doig

**Affiliations:** 1 Center of Hypothalamic Research, Department of Internal Medicine, University of Texas Southwestern Medical Center, 5323 Harry Hines Blvd., MC: 9077, Y06322A, Dallas, TX 75390-9077 USA; 2 Centre for Systems Health and Integrated Metabolic Research, Department of Biosciences, Nottingham Trent University, Nottingham, UK

**Keywords:** Glucocorticoid excess, Beta alanine, Cushing’s syndrome

## Abstract

Exogenous glucocorticoids are extensively used to combat a plethora of medical conditions. However, a sustained excess of exogenous glucocorticoids causes several adverse metabolic conditions, most notably Cushing’s Syndrome which presents with a well-defined phenotype. Herein we determine the ability of the non-proteogenic amino acid, beta alanine (BA) to mitigate the development of Cushing’s Syndrome. Female C57BL/6J mice were treated with high dose corticosterone or a vehicle control with or without BA for 3 weeks. Indirect calorimetry was conducted during the final week of treatment and glucose tolerance was assessed upon completion. BA partially attenuated the development of the Cushingoid phenotype with a reduction in central adipose accumulation, bodyweight gain and trended reduction in liver weight. Despite this reduction, BA also reversed the apparent corticosterone induced increase in energy expenditure (EE), whilst further restricting mice to carbohydrate utilisation. However, BA completely preserved glucose tolerance. We identify a novel therapeutic role of BA in mitigating the development of some of the symptoms associated with exogenous glucocorticoid treatment, most notably glucose intolerance. Further investigation is now required to determine the extent of BA’s protection as well as the mechanisms involved.

## Introduction

Exogenous glucocorticoids (GCs) are potent anti-inflammatories routinely prescribed for a variety of dysfunctions (Barnes 1998; Raff and Carroll 2015). Chronic exposure to excess glucocorticoids results in hypercortisolaemia and Cushing’s syndrome, the most prevalent form of Cushing’s (Raff and Carroll 2015; Barbot et al. 2020; Chaudhry and Singh 2024). Encompassing other metabolic pathologies, Cushing’s syndrome is characterised by a well-documented phenotype, demonstratable in humans and rodents (Lacroix et al. 2015; Lonser et al. 2017). Insulin resistance, hypertension, hepatic steatosis, myopathy and obesity manifest following sustained glucocorticoid excess (Schakman et al. 2008; Di Dalmazi et al. 2012; Goodwin and Geller 2012; Abraham et al. 2013; Woods et al. 2015; Morgan et al. 2016). Recently, an elevated metabolic rate and an increased reliance on carbohydrate utilisation have also been identified as part of the phenotype in mice (Heaselgrave et al. 2024). Whilst the presentation of symptoms is similar between males and females, severity of symptoms maybe be considered sex dependent (Kroon et al. 2020; Kaikaew et al. 2019; Gasparini et al. 2019) with exacerbated effects observed in female mice (Heaselgrave et al. 2024). Cushing’s syndrome elevates mortality risk, even if successfully resolved (Barbot et al. 2020; Lacroix et al. 2015; Oray et al. 2016; Hakami et al. 2021). Therefore, there is a need to identify treatments that dampen detrimental effects and mitigate the development of Cushing’s syndrome.

Carnosine is a histidine containing dipeptide that is endogenously produced and can be exogenously supplemented in humans (Trexler et al. 2015). Carnosine’s anti-inflammatory and antioxidant properties as well as its reported enhancement of insulin sensitivity (Jukic et al. 2021; Albrecht et al. 2017) make it a potential tool for the protection against symptoms seen in Cushing’s syndrome (Spiers et al. 2014; Cruz-Topete and Cidlowski 2015). In fact, some evidence suggests it can combat glucocorticoid induced skeletal muscle atrophy in vitro and in vivo (Rahman et al. 2024, 2025). However, potential benefit of carnosine and viability of supplementation is hampered by the high levels the carnosine degradation enzyme, carnosinase (CN1) (Bellia et al. 2014) in human serum. As an alternative approach, carnosine analogues resistant to CN1 function have had some success, but their efficacy has been limited. A rate limiting precursor in the endogenous formation of carnosine is the non-proteogenic amino acid, beta alanine (BA) (Trexler et al. 2015). Safe for oral supplementation and bioavailable within mammals (de Jager et al. 2023; Trexler et al. 2015), BA is known to be resistant to CN1 hydrolysis and has increased bioavailability in humans in comparison to carnosine (de Jager et al. 2023). Despite studies examining BA directly being few, a number demonstrate similar benefits to carnosine and clear potential for metabolic improvement in mammals (Schnuck et al. 2016; Black et al. 2018; Matthews et al. 2021).

Therefore, using an established rodent model of sustained glucocorticoid excess (Morgan et al. 2014, Fenton et al. 2019; Heaselgrave et al. 2024), we aimed to establish if concurrent supplementation with BA can mitigate the metabolic development of Cushing’s syndrome in female mice. We hypothesise that beta-alanine may recapitulate some of the beneficial metabolic effects observed with carnosine (Albrecht et al. 2017; Matthews et al. 2021; Jukic et al. 2021; Rahman et al. 2024, 2025), particularly improvements in glucose homeostasis, thereby mitigating a key metabolic features of glucocorticoid excess. Mice were fed corticosterone (100 mg/l) spiked drinking water +/- BA (12 g/l) or vehicle control, *ad libitum* for 3-weeks. Mice were assessed using high-resolution indirect calorimetry and glucose tolerance testing.

## Materials and methods

### Animals and treatments

Female C57BL/6J (wild type, WT) mice were purchased from Charles River, UK. Mice were group-housed in sex and litter matched cages in groups of 2–4. Cages were kept at a standard temperature (22 °C) and humidity-controlled environment with a uniform 12 h light/dark cycle. Mice were individually housed during the final 120 h of indirect calorimetry assessment. Throughout mice were provided nesting material with food and water available *ad libitum*. Mice were provided a standard chow diet (EURodent Diet 14%, Labdiet, St Louis, Missouri, USA) but water differed depending on treatment group. Treatment lasted for 3 weeks and utilised an established protocol known to induce a phenotype typical of sustained glucocorticoid excess (Morgan et al. 2014, Fenton et al. 2019). Drinking water containing 100 mg/L corticosterone (Sigma-Aldrich, St Louis, Missouri, US), 12 g/L BA (Sigma-Aldrich, St Louis, Missouri, US) 100 mg/L corticosterone plus 12 g/L BA or a vehicle control (0.6% ethanol) was given *ad libitum*. All mice were 10 weeks of age at the start of treatment. Mice were culled following glucose tolerance testing which occurred immediately upon completion of indirect calorimetry assessment. All animal procedures were conducted in accordance with UK Home Office regulations, UK Animals (Scientific Procedures) Act 1986 and approved locally by Nottingham Trent University AWERB committees under the project license number PP7215212.

### Indirect calorimetry

Initial indirect calorimetry was performed during the final week of treatment using a Sable Promethion 16-cage system (Sable Systems International, Las Vegas, USA). Mice were group housed for 48 h in the Promethion to acclimatise. Mice were then individually housed for 120 h within the Promethion. The first 24 h for acclimation to isolation, then 96 h undisturbed for data collection, of which only the final 48 h were used for analysis. Energy expenditure (EE), oxygen consumption, carbon dioxide production and the respiratory exchange ratio (RER) were all measured. Physical activity was also measured.

### Glucose tolerance test

Glucose tolerance was assessed following completion of indirect calorimetry assessment which was at the end of the 3-week treatment period. Mice were fasted for 6 h before blood glucose was measured from a vein on the rear foot using a glucometer (Accu-Chek, Roche) at 0, 15, 30, 60, 90 and 120 min post glucose intraperitoneal injection (2 g/kg).

### Statistical analysis

Statistical significance was tested for using GraphPad Prism version 9 (GraphPad Software, LLC) as well as CalR (version 1.3) (Mina et al. 2018). Two-way ANOVAs, followed by Tukey’s multiple comparisons test, were used to analyse all indirect calorimetry measures, glucose tolerance and physical characteristics. Energy expenditure was additionally analysed using analysis of covariance (ANCOVA), with body weight included as a covariate. Interaction between body weight and treatment group was first assessed, and where no significant interaction was detected, a common slope model was applied. Group (intercept) differences were then evaluated. Data is presented as mean ± SEM throughout.

## Results

### Beta-alanine partially attenuates the development of a Cushingoid phenotype

**Fig. 1:**
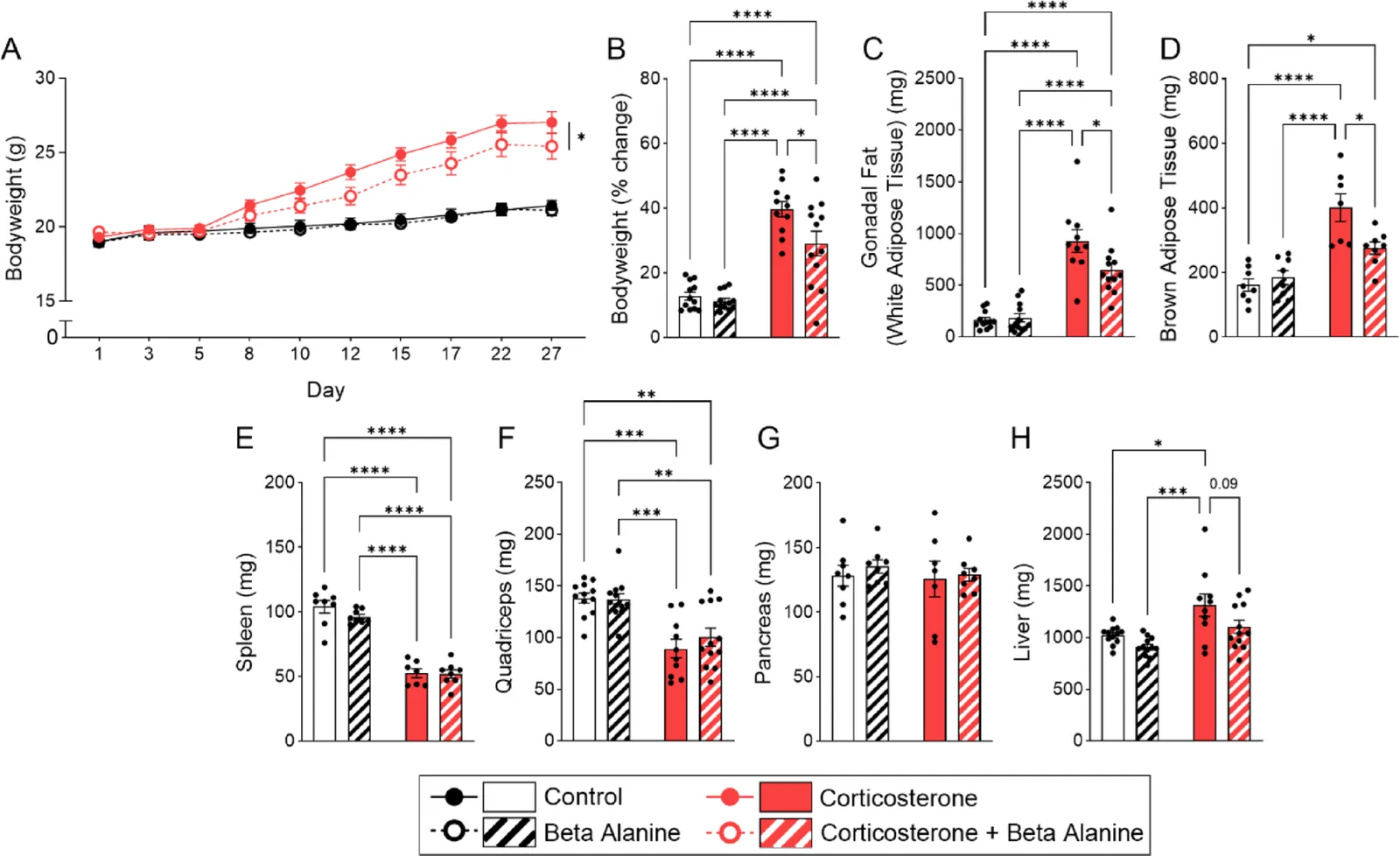
Beta-alanine (BA) partially prevents corticosterone induced weight gain and central adiposity, but not skeletal muscle or spleen atrophy. **A** Bodyweight change over the course of treatment. **B** Percentage bodyweight change following treatment. **C** Gonadal fat weight post treatment. **D** Brown adipose tissue weight post treatment. **E** Spleen weight post treatment. **F** Quadriceps weight post treatment. **G** Pancreas weight post treatment. **H** Liver weight post treatment. Line graph are presented as mean ± SEM, *n* = 7–8. Scatter plots represent are presented as mean with individual values ± SEM, *n* = 8–12. * significantly different. *p<.05, ** *p*<.01, *** *p*<.001, **** *p*<.0001

We demonstrate recapitulation of the corticosterone spiked drinking water phenotype with an increase in body weight in corticosterone fed mice (Fig. 1A and B). BA spiked drinking water alone compared to vehicle control mice showed no significant change in body weight (Fig. 1A and B). However, BA in combination with corticosterone attenuated the bodyweight increase seen in corticosterone alone (Fig. 1A and B). Despite this, the body weight of corticosterone and BA treated mice was still increased compared to the control cohort (Fig. 1A and B).

Chronic glucocorticoid exposure drives central adiposity, an effect confirmed in corticosterone fed mice. Examination of the gonadal fat pad revealed corticosterone + BA reduced adipose accumulation compared to corticosterone alone, whilst remaining elevated compared to control (Fig. 1C). The corticosterone + BA cohort also accumulated significantly less adipose tissue around their brown adipose tissues when compared to the corticosterone alone treatment (Fig. 1D).

Amongst the well characterised impacts of chronic glucocorticoid exposure is a decline in spleen and skeletal muscle mass. Concomitant with anti-inflammatory impacts of glucocorticoids there is atrophy of the spleen in both corticosterone and corticosterone + beta-alanine treated mice (Fig. 1E). Additionally, we measure a significant decrease in muscle mass alongside glucocorticoid exposure. We see no change in the efficacy of this effect when BA is present (Fig. 1F).

Other tissues remain unaffected by both corticosterone and BA alone or in combination. Pancreatic weights are unchanged by any treatment (Fig. 1G). However, liver weight trended downwards in corticosterone + BA mice compared to corticosterone alone (*p* = .09), returning to levels comparable with control mice (Fig. 1H).

### Beta-alanine alters the impact of corticosterone on metabolic rate and substrate utilisation

**Fig. 2:**
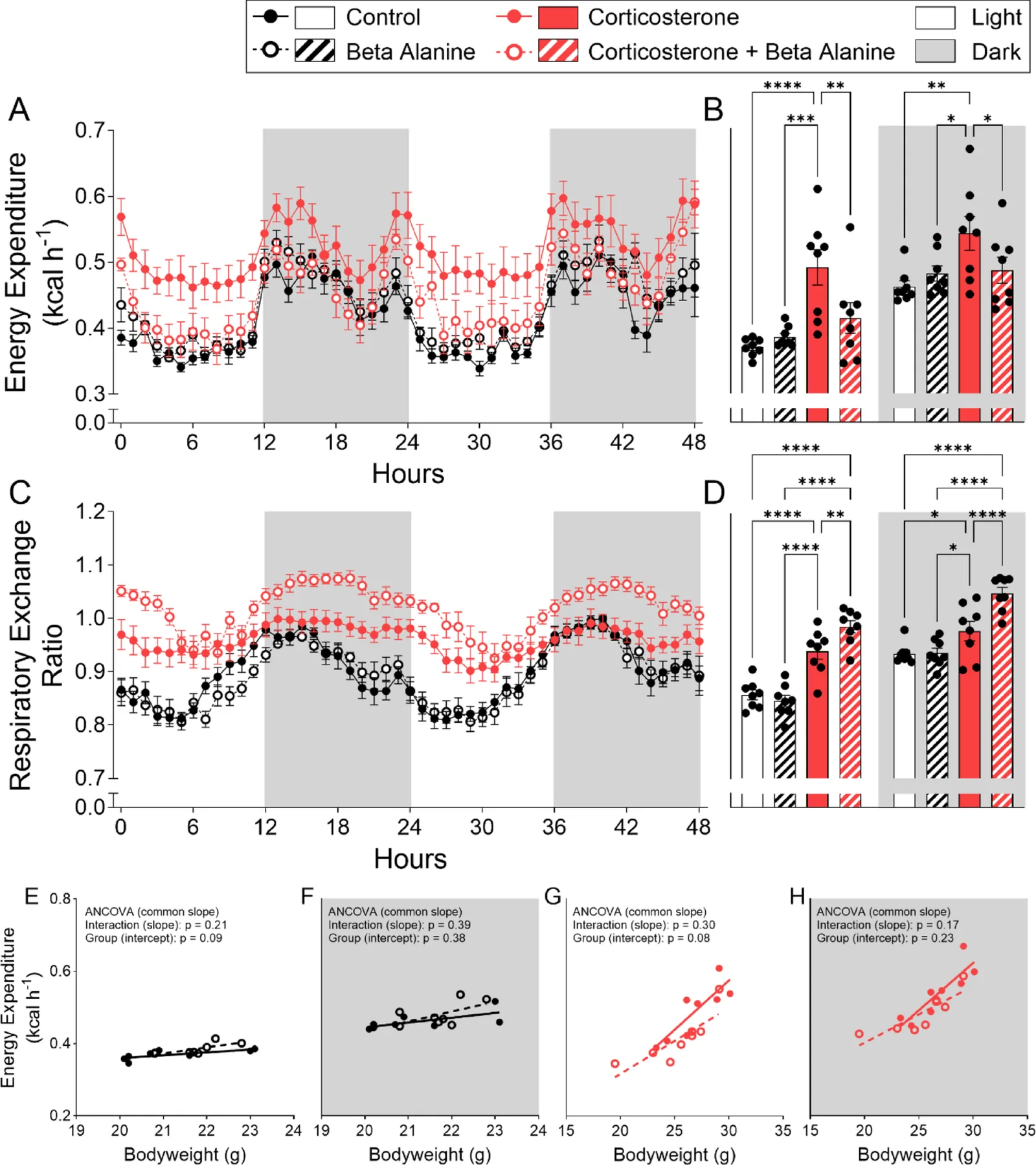
Beta-alanine prevents corticosterone induced elevation of energy expenditure but further alters the respiratory exchange ratio. **A** Energy expenditure. **B** Average light and dark phase energy expenditure. **C** Respiratory exchange ratio. **D** Average light and dark phase respiratory exchange ratio. **E**–**H** ANCOVA analysis of energy expenditure against body weight. **E** Control vs. beta-alanine during the light phase. **F** Control vs. beta-alanine during the dark phase. **G** Corticosterone vs. corticosterone + beta-alanine during the light phase. **H** Corticosterone vs. corticosterone + beta-alanine during the dark phase. ANCOVA was performed with body weight as a covariate; as no significant interaction between body weight and treatment group was detected, a common slope model was applied. Line graphs represent hourly change and are presented as mean ± SEM, *n* = 8. Scatter plots represent average light and dark vales and are presented as mean with individual values ± SEM, *n* = 8. * significantly different. *p<.05, ** *p*<.01, *** *p*<.001, **** *p*<.0001

Glucocorticoid excess has been shown to alter metabolic rate in mice (Heaselgrave et al. 2024). In agreement with this, we found corticosterone treated mice displayed an apparent increase in energy expenditure (EE), a primary measure of metabolic rate, during both the light and dark phases (Fig. 2A and B). We also find that BA alone did not perturb metabolic rate (Fig. 2A and B). However, BA concomitant with corticosterone normalised EE comparable to control treated mice, mitigating the apparent impact of corticosterone excess (Fig. 2A and B), while preserving the expected positive relationship between bodyweight and EE (Corrigan et al. 2020). To account for the influence of body weight on EE, EE was analysed using ANCOVA with body weight as a covariate. No significant interaction or group effects were detected, indicating that apparent differences in in EE were likely driven by corticosterone or beta alanine induced bodyweight effects.

**Fig. 3:**
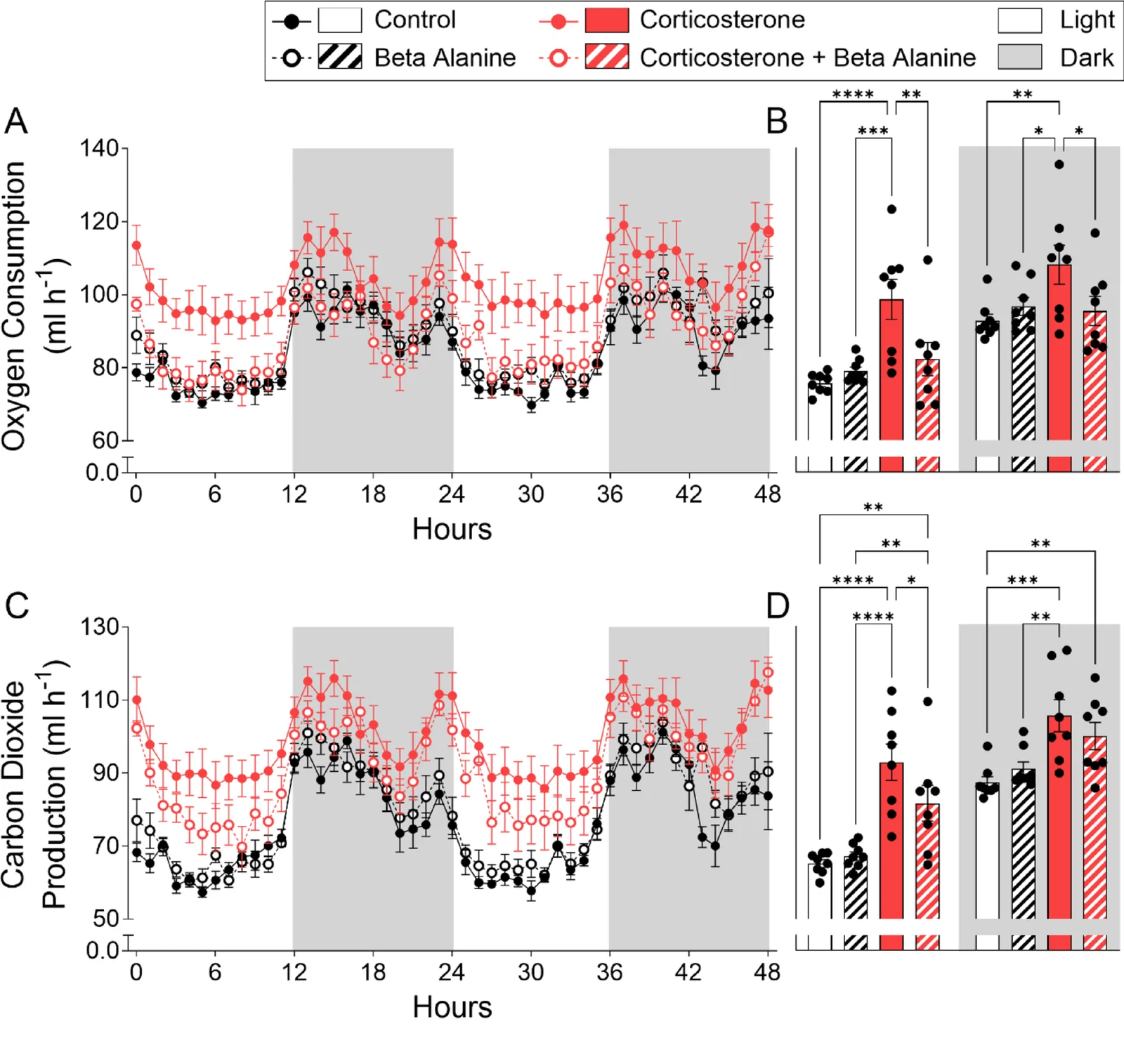
Beta-alanine prevents corticosterone induced elevation of oxygen consumption and partial prevents elevation of carbon dioxide production. **A** Oxygen consumption. **B** Average light and dark phase oxygen consumption. **C** Carbon dioxide production. **D** Average light and dark phase carbon dioxide production. Line graphs represent hourly change and are presented as mean ± SEM, *n* = 8. Scatter plots represent average light and dark vales and are presented as mean with individual values ± SEM, *n* = 8. * significantly different. *p<.05, ** *p*<.01, *** *p*<.001, **** *p*<.0001

Respiratory exchange ratio (RER) is a measure of substrate utilisation based on CO_2_ and O_2_ exchange. Consistent with excess exposure to glucocorticoids we see that corticosterone treatment flattened the natural diurnal pattern (RER), forcing mice to maintain a higher value close to, or in excess of 1.0 during both the light and dark phases, indicative of sustained carbohydrate utilisation (Fig. 2C and D). We also see that BA alone does not impact the normal fluctuations between lipid and carbohydrates as energy sources (Fig. 2C and D). However, BA in combination with corticosterone further elevates RER beyond corticosterone alone (Fig. 2C and D), likely preventing mice from utilising lipids as a fuel source. From assessment of the constituent parts of RER, O_2_ consumption and CO_2_ production, we identify that corticosterone alteration of RER is driven by an elevation of both during the light and dark phases, with a greater increase in CO_2_ production (Fig. 3A–D). Yet, whilst BA alone had no effect on either (Fig. 3A-D), BA in combination with corticosterone fully prevented an increase in O_2_ consumption (Fig. 3A and B) whilst attenuating, but not preventing, the elevation in CO_2_ production (Fig. 3C and D). These data therefore suggest that the shift in RER is a consequence of the decreased O_2_ consumption rather than increased CO_2_ production.

Therefore, in agreement with other studies (Heaselgrave et al. 2024), corticosterone exposure was associated with a greater reliance on carbohydrate oxidation. However, this pattern may reflect altered feeding behaviour and carbohydrate availability rather than a true impairment in metabolic flexibility. These findings also suggest that, in the context of glucocorticoid excess, beta-alanine may help preserve overall metabolic rate, while further reinforcing a preference for carbohydrate utilisation rather than the typical shift toward lipid oxidation.

### Beta-alanine does not alter corticosterone induced activity

**Fig. 4:**
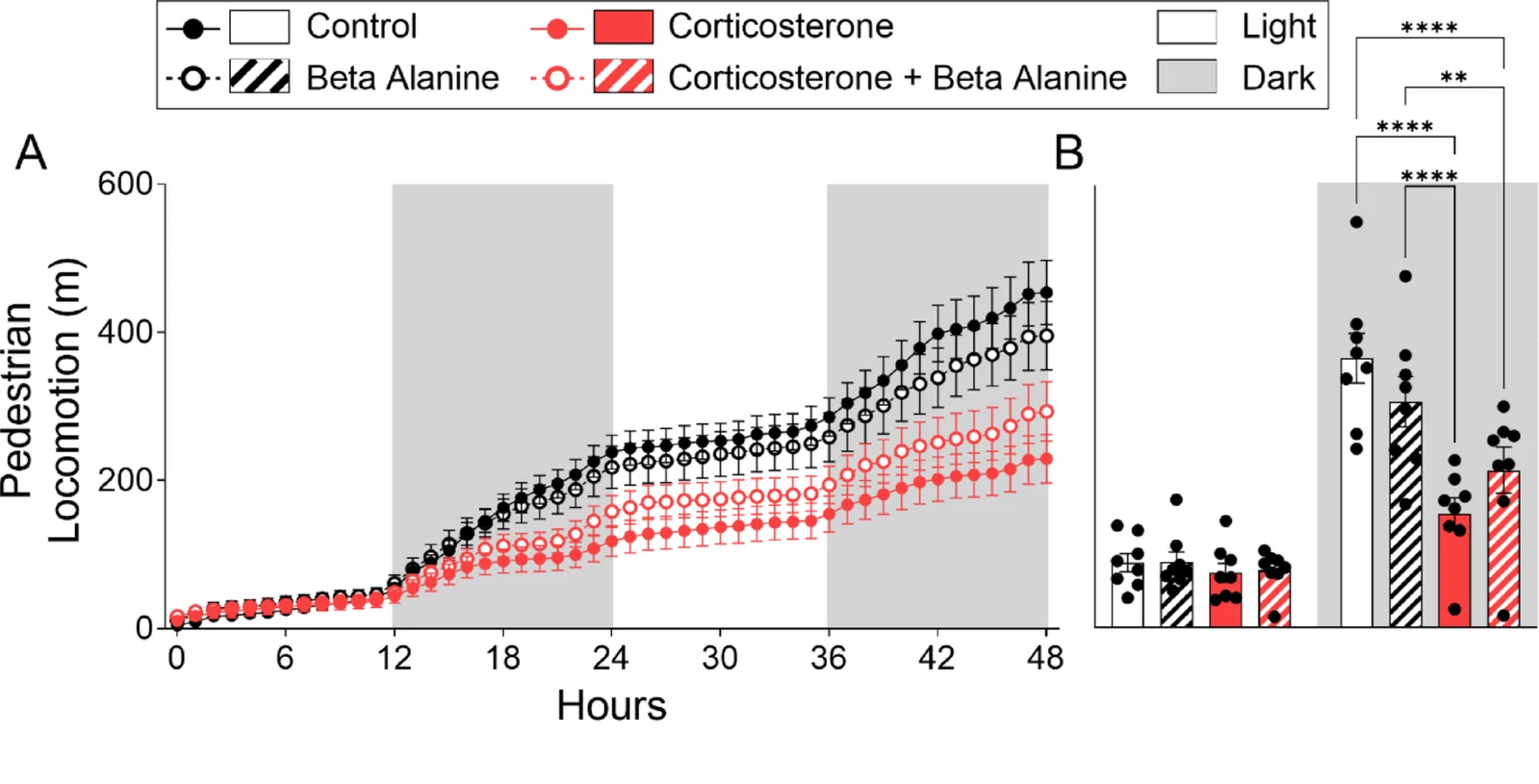
Beta-alanine does not prevent corticosterone induced inactivity. **A** Cumulative locomotion. **B** Average light and dark phase locomotion. Line graph represents hourly change and are presented as mean ± SEM, *n* = 7–8. Scatter plot represents average light and dark vales and are presented as mean with individual values ± SEM, *n* = 7–8. * significantly different. *p<.05, ** *p*<.01, *** *p*<.001, **** *p*<.0001

Glucocorticoid excess has been shown to promote inactivity (Heaselgrave et al. 2024). We further confirmed this as corticosterone significantly reduced physical activity, measured as pedestrian locomotion (meters covered) (Fig. 4A and B). BA alone produced movement data no different from control, whilst BA alongside corticosterone resulted in the same inactivity as corticosterone alone (Fig. 4A and B). Therefore, beta-alanine has no effect over physical activity.

### Beta-alanine restores glucose tolerance in conditions of glucocorticoid excess

**Fig. 5:**
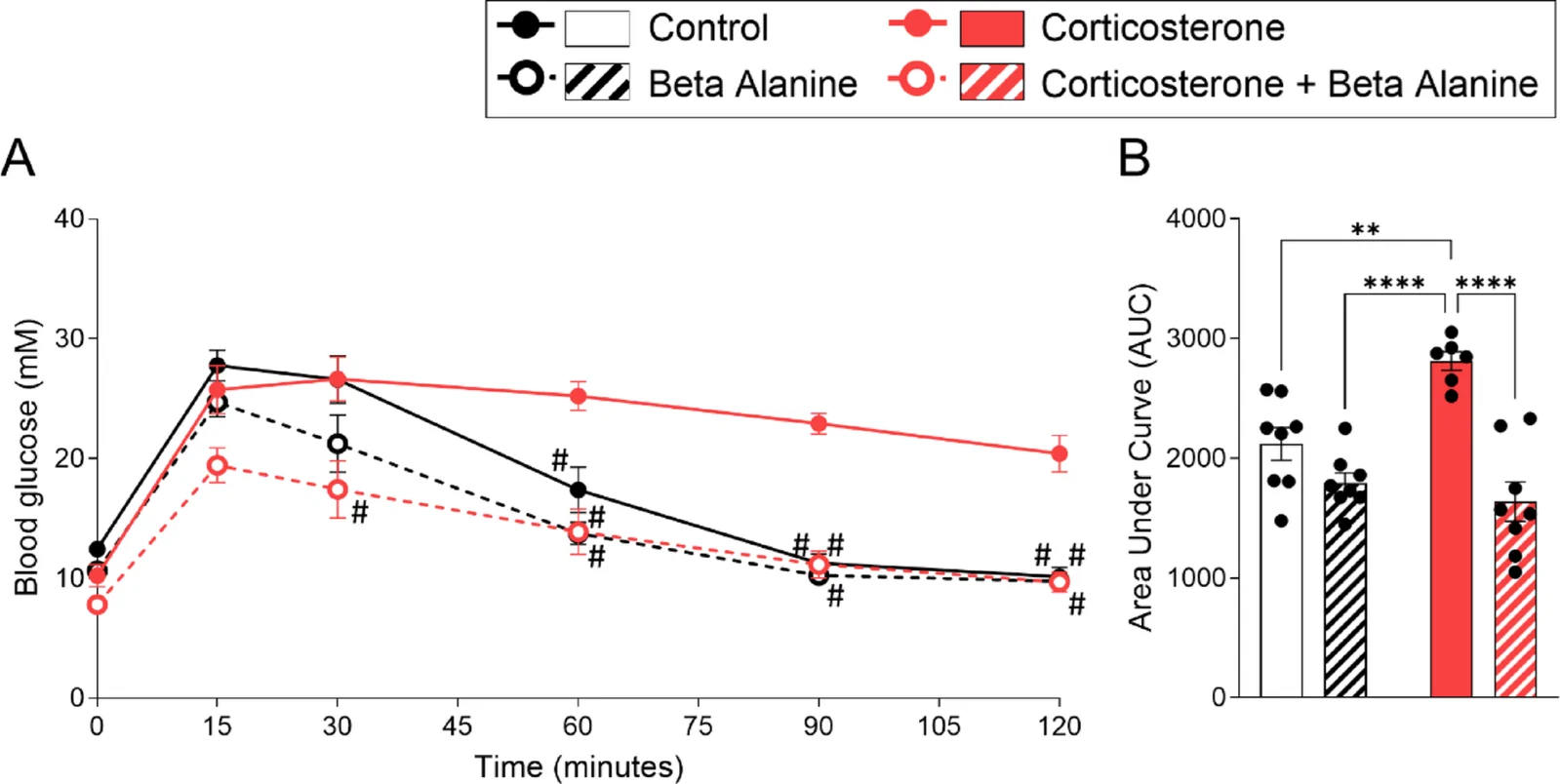
Beta-alanine prevents corticosterone induced glucose intolerance. **A** Blood glucose. **B** Area under the curve. Line graph represent change over time and are presented as mean ± SEM, *n* = 6–8. Scatter plots represent average values and are presented as mean with individual values ± SEM, *n* = 6–8. # significantly different to corticosterone, * significantly different. *p<.05, ** *p*<.01, *** *p*<.001, **** *p*<.0001

To establish the impact of beta-alanine over glucose homeostasis during glucocorticoid excess we performed glucose tolerance tests in our cohorts. Glucose disposal is lost during chronic glucocorticoid exposure. This was confirmed by us (Fig. 5A and B). Interestingly, we measured a significant recovery in the glucose handling of mice exposed to corticosterone + beta-alanine, despite BA alone having no effect (Fig. 5A and B). In fact, corticosterone + BA treated mice had significantly increased capabilities of glucose disposal (also shown by area under curve) compared to corticosterone treated and control treated mice (Fig. 5A and B).

## Discussion

Beta-alanine is bioavailable to humans and rodents and is proven to contribute towards serum carnosine levels (de Jager et al. 2023; Trexler et al. 2015). Therefore, in this study we explored the potential of beta-alanine to mitigate the many metabolic dysfunctions generated by chronic exposure to the steroid hormone class, glucocorticoids. As one of the most widely prescribed medicines its detrimental off target effects and the elevation of mortality represents a growing challenge. We used an established model of glucocorticoid excess (Morgan et al. 2014, Fenton et al. 2019) and combined it with beta-alanine spiked drinking water. This enabled exposure *ad libitum* to both compounds simultaneously.

We find corticosterone induced effects consistent with other studies (Peckett et al. 2011; Gasparini et al. 2016; Schakman et al. 2013; Sato et al. 2018; Tataranni et al. 1996; Sefton et al. 2016; Heaselgrave et al. 2024). We also find BA treatment in an otherwise healthy system to have no additive benefits, in agreement with other (Mong et al. 2011; Moreto et al. 2024). However, when supplied to a metabolically compromised system, in this case one with an excess of circulating glucocorticoids, we demonstrate clear therapeutic potential as BA partially attenuated the development of characteristics synonymous with Cushing’s syndrome in female mice. Notably BA attenuates corticosterone induced central adiposity and bodyweight gain. This is despite an apparent normalisation of EE and further increase in the RER, indicating a reduction in calorie usage and lipid utilisation. It also indicates that BA simultaneously protects and disrupts metabolic flexibility in the presence of excess exogenous glucocorticoids. Interestingly, glucose tolerance is preserved by BA, indicating complete protection against corticosterone induced diabetes. However, BA falls short of a global impact as other symptoms of Cushing’s syndrome including spleen atrophy and physical inactivity, still present. Skeletal muscle atrophy also still presented, however as this assessment relies on the weights of a single muscle, inclusion of comprehensive body composition analysis would further reinforce this finding.

Due to the novelty of data identified in this study the mechanistic drivers of BA’s impact are undetermined. A global mechanism known to offer protection against the metabolic consequences of exogenous glucocorticoid excess involves the inhibition, or knock out, of the enzyme 11β-hydroxysteroid dehydrogenase type 1 (11β-HSD1) (Tomlinson and Stewart 2001; Morgan et al. 2014; Heaselgrave et al. 2024). However, it appears unlikely BA inhibits the activity of 11β-HSD1 or stimulates a comparable global mechanism as the protection it offers is not complete. Therefore, alternate explanations must presently be considered. With regards to the attenuation of adipose tissue accumulation and bodyweight increase, an effect seen with carnosine and high-fat diet fed rodents (Aldini et al. 2011; Anderson et al. 2018; Mong et al. 2011), the underlying mechanism is not immediately apparent. However, this study determines that it is not a result of increased calorie expenditure or lipid utilisation as BA decreased EE and further shifted the RER away from lipid metabolism in corticosterone fed mice. It is plausible BA inhibits fat absorption as it might act as an inhibitor of pancreatic lipases (Emimmal 2019). However, this is uncertain as carnosine does not increase faecal lipid content in high-fat diet (HFD) fed mice (Mong et al. 2011) which would be expected if mice were eating the same volume as seen in the present study. Therefore, it remains probable BA alters adipose tissue formation, however evidence to confirm this is currently lacking. The reason for normalised EE in is also unclear. Beta-alanine attenuation of corticosterone induced bodyweight increase is potentially a contributing factor, aligning with the principle that an animal of reduced mass has a reduced energy cost associated with its actions (Corrigan et al. 2020). However, as the bodyweight of mice treated with corticosterone and BA remains elevated compared to controls, changes in bodyweight are unlikely to be the sole explanation. Other factors to consider include activity (van Baak 1999), which can be discounted as BA does not alter corticosterone induced inactivity; and brown adipose tissue (BAT) thermogenesis (Cannon and Nedergaard 2004). However, BAT thermogenesis is already reported suppressed in rodents exposed to glucocorticoid excess (Doig et al. 2017, Poggioli et al. 2013) and there is an absence of evidence to suggest BA causes further suppression needed to lower EE. Like EE, the exacerbation of the corticosterone induced shift in RER by BA is undetermined. Possible explanations for an RER in excess of 1.0 include bicarbonate buffering and increased de novo lipogenesis (DNL) (Robergs et al. 2004; Solinas et al. 2015). The former can be discounted as this is characterized by an increase in carbon dioxide production (Robergs et al. 2004), the opposite effect to that observed in this study. In fact, we determine that the greater shift in RER is instead a result of a decrease in oxygen consumption in corticosterone and BA treated mice. However, an explanation for this decrease is also lacking. As for DNL, it too is unlikely responsible for elevating RER as corticosterone treated mice supplemented with BA accumulated less adipose tissue and therefore appear to synthesize less fat. This is supported by evidence that carnosine supplementation can attenuate HFD inducement of lipogenesis in mice (Mong et al. 2011). It should also be noted that substrate oxidation estimates derived from indirect calorimetry may be modestly influenced by unmeasured nitrogen balance (Frayn 1983). Thus, nitrogen provided by BA in drinking water could theoretically influence these calculations; however, BA alone did not alter RER and water intake did not differ significantly between groups, suggesting this effect is likely minimal. It should also be noted that energy intake was not directly measured in this study. As such, differences in food consumption between groups cannot be excluded as a contributing factor to the observed metabolic phenotype. Additionally, circulating serum markers were not assessed, limiting mechanistic insight into hormonal and metabolic changes underlying these effects. The final finding to explain is BA protection of glucose tolerance in corticosterone fed mice, an effect previously seen in mice fed a HFD with carnosine (Moreto et al. 2024). Unlike many of the other findings strong evidence for a responsible mechanism exists. Both BA and carnosine supplementation have been shown to promote pancreatic β-cell insulin secretion and tissue insulin sensitivity (Albrecht et al. 2017; Jukic et al. 2021; Matthews et al. 2021), two variables that would otherwise be negatively impacted by glucocorticoid excess (Di Dalmazi et al. 2012; Kaikaew et al. 2019). It remains plausible that the antioxidant and anti-inflammatory properties of BA (Jukic et al. 2021) might also directly or indirectly enable protection against glucocorticoid excess (Anderson et al. 2018), however at present this cannot be confirmed.

## Conclusion

In conclusion, this study identifies therapeutic potential of BA for combatting some of the metabolic complications of sustained exogenous glucocorticoid treatment. However, it also raises several questions, about the underlying mechanisms. Whilst this study displays the ability of BA to mitigate the development of some of the features Cushing’s syndrome it does not explore whether BA would be of benefit to those who already have the condition, or those with endogenous Cushing’s syndrome or Cushing’s disease. Additionally, several other symptoms remain untested and whilst there is evidence BA may counter symptoms such as osteoporosis or hepatic steatosis (Anderson et al. 2018; Mong et al. 2011; Kishi et al. 1994), further research exploring this, as well as the effects of BA on substrate utilisation under conditions of glucocorticoid excess, is required.

## Data Availability

All data generated or analysed during this study are included in this article. Raw data are available from the corresponding author upon reasonable request.All data were generated as described in the Methods section of this manuscript. Data were recorded digitally and analysed using GraphPad Prism (v9) and CalR (v1.3). Statistical analyses were performed as detailed in the manuscript.
